# Endoscopic Resection of Large Non-Pedunculated Colonic Polyps Without Submucosal Injection Is Safe and Effective with Adequate Technique

**DOI:** 10.3390/jcm14020642

**Published:** 2025-01-20

**Authors:** Melis Gokce Celdir, Gilles Jadd Hoilat, Alp Serhat Kahveci, Rami El Abiad, Henning Gerke

**Affiliations:** Gastroenterology and Hepatology, University of Iowa Hospitals and Clinics, Iowa City, IA 52242, USA; gilles-hoilat@uiowa.edu (G.J.H.); alp-kahveci@uiowa.edu (A.S.K.); rami-elabiad@uiowa.edu (R.E.A.); henning-gerke@uiowa.edu (H.G.)

**Keywords:** endoscopic mucosal resection, large colorectal polyps, lift polypectomy

## Abstract

**Background/Objectives**: Endoscopic resection with lift polypectomy using submucosal injection (SI) for large non-pedunculated colorectal polyps is recommended to facilitate complete mucosal resection and decrease the risk of perforation; however, there are no studies comparing the safety and efficacy of large polypectomies with and without lift polypectomy. We aimed to evaluate the feasibility and safety of the polypectomy technique without SI compared to the routine use of SI. **Methods**: We performed a single tertiary center retrospective study evaluating all consecutive large non-pedunculated colorectal polyps (≥20 mm) referred to expert endoscopists in polypectomy from 2018 through 2021. We collected and analyzed data on demographics, polyp characteristics, resection technique, complications, and polyp recurrence in 6–12 months. **Results**: In 200 large non-pedunculated colonic polyp resections, 110 (55%) were performed with SI. The median polyp size was slightly larger in SI (30 mm IQR [20, 40] vs. 25 mm IQR [20, 30] in no-SI, *p* < 0.05), with a range of 20 to 130 mm. There were no differences in delayed bleeding rates. No perforation was noted in the no-SI group, and five perforations occurred in the SI group, without a statistically significant difference between groups. There was no statistically significant difference in the polyp recurrence rate at 6–12 months between the no-SI and SI groups (12% vs. 8% in no-SI vs. SI, respectively, *p* = 0.48). **Conclusions**: Complete removal of large non-pedunculated polyps without SI is feasible and safe in this large series. This approach had similar rates of clinically significant post-polypectomy bleeding and a non-significant difference in perforation rate compared to polyp resection with routine use of SI. Randomized trials are warranted to further assess the safety and efficacy of this approach.

## 1. Introduction

An adequate polypectomy technique is crucial for complete and safe polyp resection. Due to the paucity of evidence on the superiority of specific methods, polypectomy techniques vary in clinical practice based on physicians’ training and expertise. Gastrointestinal societies advise lift polypectomy to remove all large, non-pedunculated polyps with a conditional recommendation supported by moderate-quality evidence [1,2,3]. This recommendation is based on the theoretical advantage that SI decreases the risk of perforation, thermal injury, and bleeding by providing a submucosal cushion with the colloid lifting agent. Additionally, the dye mixed with the lifting agents highlights the submucosal space and may aid in delineating the borders of the polyp, further facilitating complete removal [4]. The guideline recommendations for lift polypectomy are based on expert recommendations [3]. Recent reports have conveyed that underwater endoscopic mucosal resection (EMR) without lift polypectomy is safe and effective [1,3,5,6]. We postulate that the principles of polypectomy without lifting also apply in a gas-filled colonic lumen. A safe resection without lift polypectomy does not necessarily require water immersion, and lifting is not needed to prevent colonic perforation. Before polypectomy, sufficient colonic decompression causes submucosal involution. This allows the lesion to pull away from the muscularis propria while the lesion and adjacent mucosa assume a polypoid configuration. This phenomenon creates a protuberance that would otherwise need lifting. Therefore, the capture of the mucosa away from the muscularis propria enables a safer polypectomy without a need for lift polypectomy. To date, no clinical studies on humans have compared the effectiveness and safety of the hot snare resection technique with and without SI in large non-pedunculated polyps.

In this feasibility and safety study, we aimed to compare the outcomes of hot snare resections of large non-pedunculated polyps with and without SI using a retrospective cohort of patients who were referred to a large tertiary care center for removal of colorectal polyps by experienced expert endoscopists in large polyp resections. We hypothesized that adequate colon decompression prior to hot snaring snare polypectomy of large non-pedunculated polyps without SI is safe and effective.

## 2. Materials and Methods

We performed a retrospective review of endoscopic removal of all large colorectal polyps performed by two expert endoscopists at a tertiary referral center between January 2018 and December 2021. One of the endoscopists preferred submucosal injection, while the other preferred a technique without injection. Patients were either referred for expert polyp removal or were found to have large, non-pedunculated polyps when the expert endoscopist performed the surveillance colonoscopy. The inclusion criteria for the cohort were (1) non-pedunculated lesions, (2) with benign, non-invasive histology, and (3) of a size equal to or larger than 2 cm. Polyps were excluded if an invasive malignancy was identified based on optical diagnosis or histopathology. We collected data on the demographics; date of procedure; use of anticoagulant or antiplatelet medications; size, number, location, and histology of polyps; polypectomy technique (piecemeal/en bloc resection, post-resection endoscopic clipping, previous removal attempt); immediate and delayed complications; and presence of residual polyp at follow-up colonoscopy. The size of the polyp was determined based on the evaluation and documentation by the endoscopist in the procedure note. Intraprocedural bleeding that was controlled during the procedure was not considered a complication. We collected data on perforations identified at the time of the procedure or after the completion of the procedure. Underwater EMR cases were excluded from the analysis. Surveillance colonoscopies were performed at 6 to 12 months. Data on whether biopsies were performed based on any tissue suspicious of recurrent or residual adenoma at repeat colonoscopies were collected. Recurrence was determined based on the resection scar biopsy or pathology of the resected tissue at the previous polypectomy site at 6 to 12 months follow-up.

### 2.1. Procedures

Colonoscopies were performed with an adult high-definition colonoscope (CF-H190AL, Olympus Medical Systems, Center Valley, PA). All patients provided informed consent for the procedures. Crescent snares (“duck bill”) in sizes of 15 mm, 25 mm, or hexagonal 13 mm snares (Cook Medical) were used during polypectomies. In the SI group, a colloid lifting agent with dye was used to lift the polyp, followed by hot snare polypectomy. In the no-SI group, standard polypectomies were performed by capturing the polyp tissue after the gas was suctioned before snare closure, and then cautery snaring of the tissue was performed. In both groups, a piecemeal resection technique was used unless the entire polyp could be captured with the snare. En bloc polyp resection was not a prioritized goal.

### 2.2. Statistical Analysis

The demographic and clinical characteristics of the patients, polyp characteristics, and associated variables of the polypectomy technique were compared according to the resection technique. Complication rates and polyp recurrence were compared using the chi-square test or Fisher’s exact test as appropriate. A *p*-value of less than 0.05 was considered to indicate statistical significance. R version 4.3.3 was used for statistical analyses.

Our institution’s institutional review board approved this study.

## 3. Results

A total of 1614 colonoscopy reports were reviewed; 200 large non-pedunculated colonic polyp resections from 172 adult patients met the inclusion criteria during the study period after excluding 17 polyps that were removed with the underwater EMR technique. The mean age of patients was 70 years (SD 10.4). Table 1 shows the characteristics of the polyps. One hundred and ten (55%) polyp resections were performed with SI. The range of polyp size was 20 mm to 130 mm. On histopathology, there were 78 (39%) tubular adenomas, 69 (35%) tubulovillous adenomas, and 48 (24%) sessile serrated lesions.

The median polyp size was slightly larger in the SI group (30 mm IQR [20, 40] in SI vs. 25 mm IQR [20, 30] in no-SI, *p* < 0.05). Sessile serrated lesions were more prevalent in the SI group (32% vs. 14% in SI vs. no-SI, respectively). Clipping was utilized more frequently in the no-SI group (82% vs. 17% in no-SI vs. SI groups, respectively). No follow-up information was available in 49 (22.6%) resections. High-grade dysplasia or carcinoma in situ was found in 17 (15.9%) of the no-SI and 13 (11.8%) of the SI group (*p* = 0.46). A previous resection attempt was noted in 21% of the polypectomies. The en bloc resection rate in the no-SI and SI groups were low and not statistically significantly different (5.6% and 1.8%, respectively, *p* = 0.16)

There was no difference in delayed bleeding rates. In our case series, no perforation was noted in the no-SI group, and five perforations were noted in the SI group, without a statistically significant difference between groups. All perforations except for the one that required hemicolectomy were treated during the endoscopy with clips and managed conservatively following the procedure with bowel rest and intravenous antibiotics. A biopsy of the scar was performed in 29% and 21% of nodular polypectomy resection sites in the no-SI and SI groups, respectively. There was no difference in the polyp recurrence rate at 6–12 months of surveillance colonoscopy in the no-SI and SI groups (12% vs. 8% in no-SI vs. SI, respectively, *p* = 0.48).

## 4. Discussion

In this retrospective case series comparing the safety outcomes of large hot snare resections of non-pedunculated colorectal polyps with and without lift polypectomy with SI, we found that a polyp resection technique without lifting with SI resulted in similar rates of perforation, recurrence, and bleeding regardless of the polyp location.

Our findings show feasibility of the hot snare polypectomy technique without lifting for large non-pedunculated colorectal polyps. Currently, EMR with SI and underwater EMR are recommended for large non-pedunculated polyps by the society guidelines. Accordingly, the no-SI polypectomy with hot snare technique for large non-pedunculated colorectal polyps is not standard practice. The assumption that lift polypectomy is essential for a safe endoscopic resection is based on expert opinion, and evidence based on clinical studies is lacking. Our study reduces an evidence gap in this regard. This is the first clinical study to date on the use of lift polypectomy in comparison to no-SI for hot resection of large, non-pedunculated colorectal polyps. The lack of significant differences in safety outcomes in this retrospective feasibility study is promising. Our results negate an old dogma and warrant further investigation with prospective, controlled trials.

Polypectomy techniques for colorectal polyps have high endoscopist variation, and incomplete resection rates remain high, ranging from 6.5% to 22.7% even when the endoscopist deemed the resection as complete [7]. The primary aim of a polypectomy is to remove a polyp entirely in the safest and most efficient way [1,3]. Lift polypectomy is recommended primarily to minimize perforation and ensure complete resection [8]. Over the recent years, studies have shown that underwater EMR without lift polypectomy is an acceptable alternative for safe polyp resection [5,6,9] and triggered the question, “Is less more?” [10]. A major component of the resection method without lift polypectomy described in this case series was the technique with sufficient colon decompression prior to polypectomy. The absence of gas is a cardinal aspect of underwater EMR. Similarly, albeit sans water, colonic decompression can be achieved by simply suctioning the excess gas and involuting the lumen, thus loosening the polyp away from the muscularis propria. Subsequently, the resulting more convoluted polyp can be better captured by the snare. This is akin to the underwater EMR described by Binmoeller et al. in their initial study [5]. A decompressed colon also facilitates mucosal and submucosal involutions to become more compact while the muscularis propria remain circular, thus allowing for maximal polyp grasp [5]. This is illustrated in endoscopic ultrasound images during colonic polyp resections underwater [5]. The polyp tissue can be snared safely away from the deeper muscularis layer, minimizing the risk of deeper tissue damage by cautery and perforation. The feasibility of cold snare resection of large sessile serrated lesions without submucosal injection was evaluated in several studies using case series [11]. Our results show that by sufficiently decompressing luminal air, polypectomy is safe without lift polypectomy for large, non-pedunculated polyps with hot cautery.

Polyp resection without SI may have time and cost advantages as it eliminates SI. Lifting with SI may increase “slippage” and render polyp capture more challenging, as the lifting agent widens the area of interest when redistributing within the submucosa. In contradistinction, underwater EMR enables larger tissue purchase even with smaller-diameter snares, allowing for higher en bloc resection rates [12]. A more voluminous polyp tissue after lifting and the time spent performing SI before resection may prolong polyp resection time. Durations of procedures were not available to compare between the two groups in this retrospective study. From a cost-effectiveness perspective, the non-SI group utilized more clips in this study. This is largely explained by a difference in personal preference between the two endoscopists, of whom one performed more SI-procedures and the other one more non-SI-procedures. Whether an increased clip use is required in the non-SI-group and whether the use of SI injection can be offset by any cost savings from using fewer clips should be evaluated in further studies with a uniform protocol regarding clip use in both groups.

Furthermore, the submucosal injection may result in a painful inflammatory reaction presenting as a post-polypectomy syndrome. Cancer seeding into deeper tissue through submucosal injection in neoplastic lesions has anecdotally been described and remains a concern, although this has not been confirmed in further studies [13,14].

A primary rationale for using lift polypectomy is to minimize the perforation risk by mobilizing the mucosa away from the muscularis propria and reduce thermal injury to deeper tissue layers by creating a safe insulating cushion [15]. In addition, incorporating a dye solution facilitates the delineation of resection planes and the identification of deeper tissue injury and perforation post-resection should they occur [16]. Our experience challenges the concept that SI is indispensable for safety. We have not found differences in perforation rate in the group without SI compared to the SI group. With the increasing use of modern microprocessor-controlled electrosurgical units over the past decade, this theoretical advantage may no longer apply to the safer resection of large polyps that were once deemed unsafe to resect without lift polypectomy.

A disadvantage of the no-SI resection technique is the compromised visibility when the polyp tissue is captured with the snare. Therefore, we recommend considering delineating the borders with snare tip coagulation, especially in sessile serrated polyps, where the borders of the polyp may not be easy to recognize after a removal attempt. The recurrence rate was comparable between the SI and non-SI groups in our study and was within the published range [17,18]. This recurrence rate can be further reduced with snare tip coagulation of margin [19].

Post-polypectomy bleeding was similar between groups and within the published range.

Underwater EMR and endoscopic submucosal resection are encouraged in the most recent guideline recommendations of gastrointestinal societies, partly because of a higher en bloc resection rate with non-pedunculated lesions measuring 15 to 30 mm [1]. In our study, en bloc resection was not a goal during polypectomy, and the non-SI approach intentionally used a piecemeal technique with relatively small snares (15 mm). Therefore, our retrospective study cannot discern whether one of the two techniques is more advantageous regarding en bloc resection. Despite the increasing adoption of endoscopic submucosal resection to treat laterally spreading colorectal lesions, piecemeal EMR remains a viable first-line option. Although early recurrence is more common after piecemeal EMR than endoscopic submucosal resection, this can be easily managed endoscopically and the initial advantage of endoscopic submucosal resection disappears with subsequent surveillance [20,21]. The longer procedure times and higher complication rates with endoscopic submucosal resection may only be justified in select patients and need to be individualized on a case-by-case basis.

This study has several limitations. First, one of the two endoscopists almost exclusively performed all non-lift polypectomies. Thus, our results may be partly due to endoscopist-dependent variations in technique and not solely due to SI utilization. This also raises the possibility that the larger reported polyp size in the SI group could be due to the variation in judging the size of the polyps by the endoscopist. We are unable to explain the difference in size of polyps between the groups otherwise, since large polypectomy referrals occurred randomly to the two endoscopists. True differences in polyp size in the two groups may impact our findings of procedural complications, recurrence rate, and clip use rates for defect closure. A prospective randomized protocol is necessary to minimize this potential bias. Furthermore, clips were utilized more in the no-SI group because the endoscopist preferred to routinely use clips to prevent delayed bleeding. Given the large defect size, clipping may not have been feasible in some instances. Additionally, the follow-up was short and incomplete. Procedures were performed at a large tertiary center and may not be reproducible in a community practice setting. Due to the retrospective nature of this study, we did not have procedure duration data available to us from medical records to compare the time efficiency of the two endoscopic approaches. While these limitations prevent judging one approach superior to the other, our data demonstrate that piecemeal polypectomy of large non-pedunculated polyps can be safely done without SI.

## 5. Conclusions

The conviction that SI is a sine qua non for the safe resection of large non-pendulated polyps has recently been challenged by the introduction of underwater EMR. Our experience suggests that water immersion is not required. Adequate decompression of the luminal gas during hot snare resection of the polyp, akin to underwater EMR, may obviate the need for SI. The polypectomy technique without SI was safe and effective in this feasibility study comparing SI to no-SI in large non-pedunculated polyp resections. Given that human study data warranting SI use in polyp resection are lacking, our study results call for further larger controlled studies with equal distribution of techniques between endoscopists and similar protocols regarding clip use. A future study should also include procedure duration to compare the time and cost efficiency of the groups.

## Figures and Tables

**Table 1:** Characteristics and complications of polyp resections with and without submucosal injection.

	No Submucosal Injection n = 90	Submucosal Injection n = 110	*p*-Value
Age, y (mean (SD))	69.7 (9.4)	69.5 (11.2)	0.88
Male (%)	52 (58)	51 (46)	0.14
Previous resection attempt	18 (20)	24 (22)	0.86
En bloc resection (%)	5 (5.6)	2 (1.8)	0.16
Location (%)			
Cecum	13 (14)	30 (27)	
Ascending colon	41 (46)	47 (43)	
Transverse colon	13 (14)	23 (21)	
Descending colon	7 (8)	3 (3)	
Sigmoid colon	6 (7)	3 (3)	
Rectum	11 (10)	4 (4)	
Size, mm (median [IQR])	25 [20, 30]	30 [20, 40]	<0.05
Anticoagulant use (%)	18 (20)	13 (12)	0.16
Clip use (%)	70 (78)	16 (15)	<0.01
Pathology (%)	0.01		
Tubular adenoma	35 (39)	43 (39)	
Tubulovillous adenoma	40 (44)	29 (26)	
Sessile serrated adenoma/lesion	13 (14)	35 (32)	
Other	2 (2)	3 (3)	
High-grade dysplasia/carcinoma in situ (%)	13 (14)	13 (12)	0.74
Delayed bleeding (%)	8 (9)	5 (5)	0.34
Perforation (%)	0 (0)	5 (5)	0.11
Recurrence at 6–12 months (%)	11 (12)	9 (8)	0.48
Mucosal resection scar biopsy in follow-up	26 (29)	23 (21)	0.27

## Data Availability

Raw data are unavailable due to privacy or ethical restrictions. The data presented in this study are available on request from the corresponding author due to privacy.

## Abbreviations

The following abbreviations are used in this manuscript:

EMR	endoscopic mucosal resection
IQR	inter-quartile range
SI	submucosal injection
